# Subclonal heterogeneity sheds light on the transformation trajectory in IGLV3-21^R110^ chronic lymphocytic leukemia

**DOI:** 10.1038/s41408-022-00650-4

**Published:** 2022-03-30

**Authors:** Lisa Paschold, Donjete Simnica, Ramon Benitez Brito, Tianjiao Zhang, Christoph Schultheiß, Christine Dierks, Mascha Binder

**Affiliations:** 1 Department of Internal Medicine IV, Oncology/Hematology, Martin-Luther-University Halle-Wittenberg, Halle (Saale), Germany; 2 Department of Hematology, Inselspital, University of Bern, Bern, Switzerland

**Keywords:** Chronic lymphocytic leukaemia, Clonal selection, B cells, VDJ recombination

**Dear Editor,**

In chronic lymphocytic leukemia (CLL), a specific G-to-R replacement at the boundary of variable and constant region of the B cell receptor (BCR) light chain IGLV3-21 (IGLV3-21^R110^) facilitates homotypic BCR-BCR interactions [1–7]. While virtually all CLL BCR are prone to autonomous signaling induced by such contacts, the IGLV3-21^R110^ molecular interaction defines a distinct clinical subset of patients that show a very aggressive clinical course [8].

While we currently lack an exact mechanistic understanding of how the CLL-promoting R110 residue is generated, its localization at the boundary of variable and constant regions suggests that it may be a product of errors in double strand break repair in the process of the V-J rearrangement of the light chain. This is especially interesting since mutations in SF3B1 and ATM have been found overrepresented in IGLV3-21^R110^ expressing CLL [9, 10]. Both genes are involved in DNA damage response and repair of DNA double strand breaks that do occur in the V-J recombination process [11, 12]. This could open up the intriguing possibility that these mutations are acquired first and may subsequently prevent the repair of point mutations such as the one resulting in IGLV3-21^R110^.

If indeed IGLV3-21^R110^ was generated through dysfunctional V-J recombination that is facilitated through mutations in genes involved in DNA repair, this would require acquisition of such mutations before light chain V-J recombination e.g. at the pre-B cell stage or earlier. The error permissive environment created by such mutations would be supported by the finding of different CLL clones that undergo independent G to R exchanges in the process of V-J recombination.

To investigate this hypothesis and thereby gain insight into the transformation trajectory of IGLV3-21^R110^ CLL, we screened a cohort of 127 CLL by flow cytometry for IGLV3-21^R110^ using an IGLV3-21^R110^ specific antibody (Fig. 1A). Twelve IGLV3-21^R110^ expressing CLL cases were identified making up 9.4% of the overall CLL cohort. These were studied in greater detail using next-generation sequencing (NGS) of immunoglobulin gene rearrangements on flow cytometry sorted and unsorted cells, evolutionary reconstruction of the CLL clone by analysis of V(D)J rearrangements and somatic hypermutation as well as genetic typing for driver gene mutations and chromosomal deletions as described in the Detailed Methods Section.

**Fig. 1:**
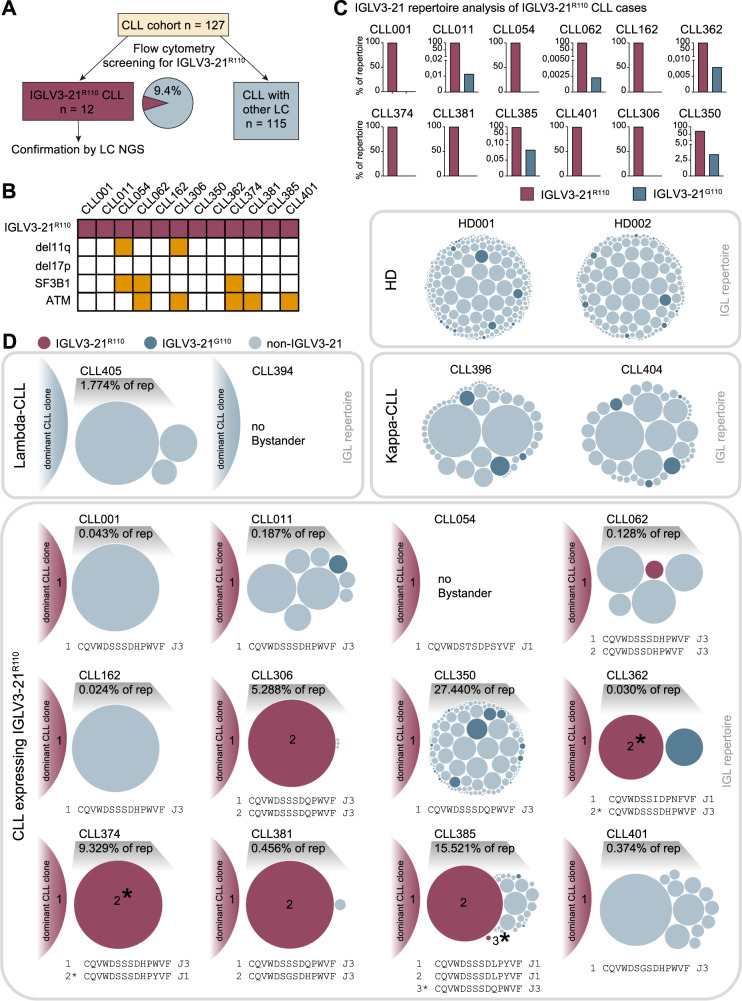
Overview of IGLV3-21 usage in the CLL cohort and subclonal landscape of IGLV3-21^R110^ expressing CLL cases. **A** Screening of the CLL cohort for IGLV3-21^R110^ via flow cytometry and confirmation of results by next-generation sequencing (NGS) of the B cell receptor light chain (LC) repertoire. **B** Mutational profile of IGLV3-21^R110^ expressing CLL cases. **C** Analysis of the IGLV3-21 immune repertoire in patients with IGLV3-21^R110^ expressing CLL. Frequencies of IGLV3-21^G110^ and IGLV3-21^R110^ sequencing reads are plotted. **D** Clonal distribution of the IGLV immune repertoire in 12 patients with IGLV3-21^R110^ expressing CLL as well as 6 control cases with nonIGLV3-21^R110^ expressing lambda CLL, kappa CLL or healthy donors (HD). One bubble represents one IGLV clone. The area size of the bubbles is proportional to the clonal frequency except for the main CLL clone which dominates the repertoires. Cases with diverging IGLJ in IGLV3-21^R110^ bystander clones are marked with an asterisk (*).

Indeed, genetic alterations in ATM and SF3B1 that are associated with the DNA damage response and repair of DNA double strand breaks that do occur in the V-J recombination process were found overrepresented in our IGLV3-21^R110^ CLL cases thereby confirming prior evidence: [9, 10] 42% (5 of 12) of the IGLV3-21^R110^ CLL cases harbored mutations in ATM and 25% (3 of 12) in SF3B1 compared to 10–15% mutation frequency of each ATM and SF3B1 in unselected CLL (Fig. 1B).

All twelve cases were subjected to light chain NGS. As a control, two kappa and two lambda expressing non-IGLV3-21^R110^ CLL cases and two healthy individuals were sequenced. The amplification protocol did not rely on J gene primers and instead used primers annealing to the intronic regions downstream of the J genes to be able to detect position 110 with high fidelity (Supplementary Fig. 1). The NGS analysis confirmed prior antibody staining results regarding the presence or absence of the R110 residue in the main CLL clone (Fig. 1C, Supplementary Table 1). Kappa deleting element rearrangement was found in all but one IGLV3-21^R110^ case. Moreover, we detected sequences coding for the G110 residue in normal bystander B cells expressing IGLV3-21, pointing at the acquired nature of the R110 residue in the CLL clone (Fig. 1C). In half of the studied IGLV3-21^R110^ CLL cases (six of twelve), we found evidence for more than one clone carrying the R110 residue (Fig. 1D). It is well known that CLL BCR undergo clonal evolution as evidenced by ongoing somatic hypermutation of their immunoglobulin variable regions. In our dataset, however, we detected not only mutational differences between IGLV3-21^R110^ expressing clones, but also different V-J rearrangements documenting different clonal origins (Fig. 1D). To rule out the possibility that identical J genes are masked by somatic hypermutation to the point that they appear as different germline cassettes, we confirmed the clonal rearrangement in the respective cases by aligning the intron sequences following the J genes to the genome sequences (Supplementary Fig. 1). The finding of differential J gene usage of clones bearing the R110 mutation within the same patient shows that these clones must have acquired the R110 point mutation independently of each other. This, in turn, could point to an error-prone environment which would increase the chances of generating R110 in the course of a dysfunctional V-J recombination.

Case CLL374 enabled us to reconstruct its transformation trajectory. In this patient, thorough analysis of the heavy chain somatic hypermutation pattern was possible for both malignant clones since the smaller subclone made up ~10% of the repertoire. First, we confirmed that the smaller IGLV3-21^R110^ expressing clone indeed represented a CLL subclone by repetition of immunoglobulin heavy and light chain NGS on sorted CD19+/CD5+ cells (Fig. 2A). This analysis confirmed the presence of both IGLV3-21^R110^ light chains in the CD19+/CD5+ population. Moreover, this analysis showed that both malignant subclones in this patient shared the same heavy chain VDJ rearrangement, yet with a different somatic hypermutation pattern (Fig. 2B). This suggested that the CLL-determining mutations in the CLL founder clone (ATM and SF3B1) have likely occurred at the level of the pre-B cell after immunoglobulin heavy chain rearrangement (IGHV3-48/D3-10/J5) but before light chain rearrangement, while the two R110 exchanges must have been acquired independently of each other at the time of light chain recombination (IGLV3-21/J1 and IGLV3-21/J3). The early separation of the two CLL clones after heavy chain rearrangement are further supported by fully divergent somatic hypermutation trajectories in the IGHV gene (Fig. 2B, C).

**Fig. 2:**
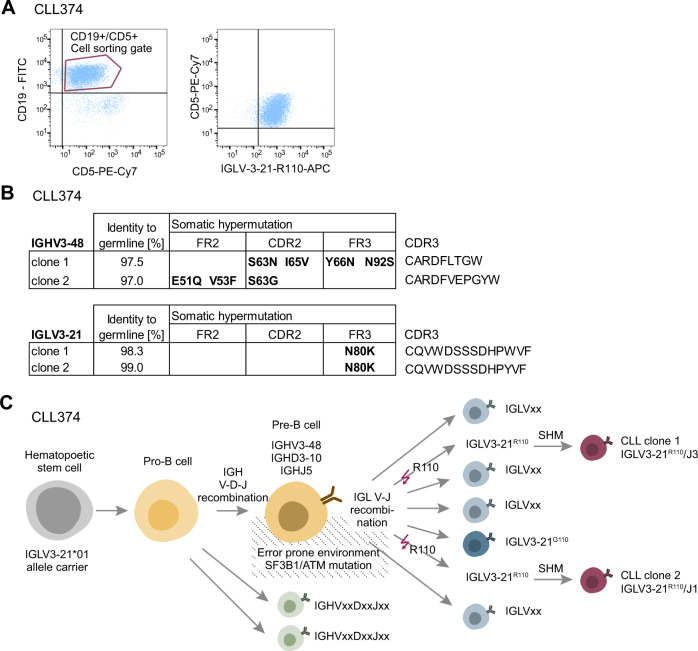
Clonal evolution in patient CLL374. **A** Exemplary flow cytometric image of one CLL case positive for expressing IGLV3-21^R110^ (CLL374). The images were created using FlowJo™ Software (BD Biosciences). The CD19 + /CD5 + cell fraction of CLL374 was sorted and subjected to heavy and light chain NGS for confirmation of CLL clone sequences. **B** Sites of somatic hypermutation in IGHV and IGLV genes of clone 1 and 2 of CLL374 with discordant IGLV-J rearrangements. Clone 1 and 2 were found in bulk sequencing of genomic DNA isolated from PBMCs as well as in the CD5 + /CD19 + cell fraction sorted via FACS (**A**). **C** Schematic representation of clonal trajectories leading to different CLL subclones in patient CLL374.

These data are surprising and generate fascinating insight into the clonal evolution of CLL. One major new aspect is the finding that IGLV3-21^R110^ CLL is not a strictly monoclonal disease, at least if we define monoclonality by a BCR consisting of the same heavy and light chain rearrangement. Intriguingly, the different clones constituting one tumor must have developed their major driver mutation—the R110 light chain residue—in a parallel evolutionary trajectory. This supports the pronounced functional importance of the IGLV3-21^R110^ light chain as a tumor driver in this CLL subset. At the same time, this data suggest that although the mutational process is stochastic, genetic trajectories may be heavily constrained by convergence on key signaling pathways. In our setting, this highlights the robust evolutionary selection of BCR-BCR interaction and autonomous signaling.

On the other hand, this data may provide first insight—although no formal proof—why and how different clones are sent on a parallel evolutionary track in CLL patients of this subset. In case CLL374, we find both divergent light chain clones to express the same heavy chain rearrangement. Clonal diversification, therefore, very likely occurred after heavy chain rearrangement, but before light chain rearrangement. The fact that two different CLL clones emerge from the same heavy chain rearranged precursor B cell suggests that this precursor may already harbor genomic lesions conferring increased proliferative potential. The driver mutations found in CLL374 were lesions of SF3B1 and ATM that are classically associated with IGLV3-21^R110^ expressing CLL [9, 10]. In addition to increasing proliferative potential of the precursor B cell, these driver mutations may also create an error-prone environment impeding error-repair in V-J recombination. Ultimately, this may lead to accumulation of clones with G-to-R substitutions at position 110 at the boundary of light chain variable and constant regions. The possibility that mutations in SF3B1, ATM or functionally similar genes may exert their transformative role if expressed before the BCR is assembled, may be further supported by the development of CLL-like cells in a murine SF3B1 mutation/ATM deletion model [13] as well as by the increased CLL risk in humans with germline polymorphisms of ATM and those with the neurodegenerative disorder ataxia telangiectasia caused by germline loss of ATM [14, 15].

Taken together, our data shed light on the transformation trajectory in IGLV3-21^R110^ CLL. To our knowledge, our work demonstrates for the first time a branching, but functionally convergent evolution in CLL thereby emphasizing the role of IGLV3-21^R110^ as a tumor promotor involved in malignant transformation of a very aggressive CLL subset.

## Supplementary information

Detailed Methods and Supplemental Material

## References

[1] Maity PC, Bilal M, Koning MT, Young M, van Bergen CAM, Renna V (2020). IGLV3-21*01 is an inherited risk factor for CLL through the acquisition of a single-point mutation enabling autonomous BCR signaling. Proc Natl Acad Sci USA.

[2] Minici C, Gounari M, Übelhart R, Scarfò L, Dühren-von Minden M, Schneider D (2017). Distinct homotypic B-cell receptor interactions shape the outcome of chronic lymphocytic leukaemia. Nat Commun.

[3] Stamatopoulos B, Smith T, Crompot E, Pieters K, Clifford R, Mraz M (2018). The light chain IgLV3-21 defines a new poor prognostic subgroup in chronic lymphocytic leukemia: results of a multicenter study. Clin Cancer Res.

[4] Kostareli E, Sutton LA, Hadzidimitriou A, Darzentas N, Kouvatsi A, Tsaftaris A (2010). Intraclonal diversification of immunoglobulin light chains in a subset of chronic lymphocytic leukemia alludes to antigen-driven clonal evolution. Leukemia.

[5] Agathangelidis A, Darzentas N, Hadzidimitriou A, Brochet X, Murray F, Yan XJ (2012). Stereotyped B-cell receptors in one-third of chronic lymphocytic leukemia: a molecular classification with implications for targeted therapies. Blood.

[6] Agathangelidis A, Chatzidimitriou A, Gemenetzi K, Giudicelli V, Karypidou M, Plevova K (2021). Higher-order connections between stereotyped subsets: implications for improved patient classification in CLL. Blood.

[7] Gemenetzi K, Psomopoulos F, Carriles AA, Gounari M, Minici C, Plevova K (2021). Higher-order immunoglobulin repertoire restrictions in CLL: the illustrative case of stereotyped subsets 2 and 169. Blood.

[8] Nadeu F, Royo R, Clot G, Duran-Ferrer M, Navarro A, Martín S (2021). IGLV3-21R110 identifies an aggressive biological subtype of chronic lymphocytic leukemia with intermediate epigenetics. Blood..

[9] Navrkalova V, Young E, Baliakas P, Radova L, Sutton LA, Plevova K (2016). ATM mutations in major stereotyped subsets of chronic lymphocytic leukemia: enrichment in subset #2 is associated with markedly short telomeres. Haematologica.

[10] Strefford JC, Sutton LA, Baliakas P, Agathangelidis A, Malčíková J, Plevova K (2013). Distinct patterns of novel gene mutations in poor-prognostic stereotyped subsets of chronic lymphocytic leukemia: the case of SF3B1 and subset #2. Leukemia.

[11] Bredemeyer AL, Sharma GG, Huang CY, Helmink BA, Walker LM, Khor KC (2006). ATM stabilizes DNA double-strand-break complexes during V(D)J recombination. Nature.

[12] Te Raa GD, Derks IA, Navrkalova V, Skowronska A, Moerland PD, van Laar J (2015). The impact of SF3B1 mutations in CLL on the DNA-damage response. Leukemia.

[13] Yin S, Gambe RG, Sun J, Martinez AZ, Cartun ZJ, Regis FFD (2019). A murine model of chronic lymphocytic leukemia based on B cell-restricted expression of Sf3b1 mutation and Atm deletion. Cancer Cell.

[14] Speedy HE, Di Bernardo MC, Sava GP, Dyer MJ, Holroyd A, Wang Y (2014). A genome-wide association study identifies multiple susceptibility loci for chronic lymphocytic leukemia. Nat Genet.

[15] Boultwood J (2001). Ataxia telangiectasia gene mutations in leukaemia and lymphoma. J Clin Pathol.

