# Supplementary material for: Subclonal heterogeneity sheds light on the transformation trajectory in IGLV3-21R110 chronic lymphocytic leukemia
Source: Blood Cancer J. 2022 Mar 30;12(3):49. doi: 10.1038/s41408-022-00650-4 (PMC8969164; doi:10.1038/s41408-022-00650-4)
Supplement: Supplementary file 1 — Detailed Methods and Supplemental Material [file 41408_2022_650_MOESM1_ESM.docx]

**Supplementary Information**

**Methods**

Blood samples from 127 CLL patients were collected after informed consent as approved by the ethics committees of the Universities of Hamburg–Eppendorf, Freiburg and Halle-Wittenberg. IGLV3-21^R110^ expressing CLL was characterized by mutational profiling, flow cytometry, cell sorting and next-generation sequencing (NGS) of the heavy and light chain loci as previously described (1-9).

***Flow Cytometry***

The whole cohort of 127 CLL cases was analyzed for the expression of IGLV3-21^R110^ using an APC-labelled IGLV3-21^R110^-specific antibody from AVA Lifescience GmbH (ApLife^TM^ FastScreen_CLL_, Denzlingen, Germany) on a BD FACSCalibur™ instrument (BD Biosciences, Franklin Lakes, USA). Cases with more than 20% of cells within the APC gate were considered positive for IGLV3-21^R110^. Case CLL374 was additionally stained using FITC-anti-CD19 and PE-anti-CD5 antibodies (AVA Lifescience, Denzlingen, Germany). For CLL374, CD19+/CD5+ cells were sorted and collected using the BD FACSAria^TM^ II system (BD Biosciences, Franklin Lakes, USA). Sorted fractions were subjected to immunosequencing of heavy and light chain as described below.

***Targeted Analysis of SF3B1 and ATM mutations via NGS***

ATM and SF3B1 genes were screened for mutations using a QIAseq Targeted DNA Custom Panel (Qiagen, Hilden, Germany) including all exonic regions of these genes, as described in ref (8). Library preparation was performed according to the supplier’s instructions using 100 ng genomic DNA input. NGS and demultiplexing was performed on an Illumina NextSeq instrument (Illumina, San Diego, USA) as a 300-cycle dual indexed (8 nucleotides) paired-end run at an estimated read depth of 26 500 reads. Data processing and variant calling was done using the CLC workbench tool (Qiagen, Hilden, Germany).

***Analysis of chromosome deletions***

Deletions in genetic loci of genes ATM and TP53 were analyzed by fluorescent in situ hybridization (FISH). For each IGLV3-21^R110^ expressing CLL case, 100 nuclei were analyzed using probes XL ATM/11cen - Deletion Probe and XL TP53/17cen - Deletion Probe from MetaSystems Probes (Altlussheim, Germany) hybridizing to 11q22.3 and 17p13, respectively.

***Next-generation sequencing (NGS)***

The GenElute Mammalian Genomic DNA Miniprep kit (Sigma-Aldrich, St. Louis, United States) was used for isolation of genomic DNA from peripheral blood mononuclear cells (PBMNCs). Bulk IGH, IGK, IGK-Kde and IGL repertoires were obtained from 250 ng genomic DNA of PBMCs as described in refs (1, 4-7, 9). IGH amplicons were generated using BIOMED2-FR1 primer pool (10). IGK and IGK-Kde primer pools were derived from (11). The IGL primer pool was adapted from (10) to cover the complete IGLJ (FR4) region including the first nucleotide of the triplet for amino acid position 110 at the junction of IGLJ and IGLC. The sequences of the new reverse primers are (5’–3’): GTGAGACAGGCTGGG, CAAGAGCGGGGAAGG, CAACTTGGCAGGGAAAG, GGGAGACCAGGGAAG, TCACCCTAGACCCAAAAG and ACTTGGGATCTCAAAGAGG. The rearranged IGH and IGL loci were amplified in a multiplex PCR using Phusion™ High-Fidelity DNA Polymerase (Thermo Fisher Scientific, Waltham, USA). PCR amplicons were purified via agarose gel electrophoresis and subjected to a second PCR for the addition of 7-nucleotide single indices and Illumina adapter sequences. NGS and demultiplexing was performed on an Illumina MiSeq sequencer (Illumina, San Diego, USA) with a 601-cycle paired-end run and V3-chemistry.

***Data analysis and plotting***

The MiXCR framework (12) with the IMGT library (13) as reference for sequence alignment was used for clonotype assembly. Only productive reads were used and clonotypes with 5 reads or less were dropped. Amino acid position 110 was defined from nucleotide 28 of the FR4 region. Nucleotide G in this position results in wild-type G110 on protein level, nucleotide C results in variant R110. IGK-Kde reads were extracted directly from fastq files. All analyses and data plottings were performed using RStudio version 3.5.1., Graph Pad Prism version 8.3.1, Adobe Illustrator version 24.1.1 and FlowJo Software.

Bubble plots of the IGL repertoire were generated with R packages packcircles (14) and ggplot2 (15) as described in refs (2, 3). One bubble represents one IGL clonotype, which is defined as unique CDR3 nucleotide sequence (MiXCR default). Clonotypes using IGLV3-21^R110^ or IGLV3-21^G110^ are highlighted. The area size of the bubbles are proportional to clone fractions within the IGL repertoire, except for the dominant clone in CLL cases expressing lambda chain (lambda-CLL) which was excluded from plotting and schematically added afterwards due to scaling reasons.

**Tables**

**Supplementary Table 1: Sequence information and characteristics of malignant clones.**

|  |  | **IGH** | | | | **IGL** | | |  |
| --- | --- | --- | --- | --- | --- | --- | --- | --- | --- |
| **CLL case** | **Mut. status** | **IGHV** | **IGHD** | **IGHJ** | **IGH-CDR3** | **IGLV** | **IGLJ** | **IGL-CDR3** | **Subset** |
| CLL001 | M | V3-21 | D3-22 | J6 | CALDRDGMDVW | V3-21 | J3 | CQVWDSSSDHPWVF | # 2 |
| CLL011 | M | V3-21 | NA | J6 | CARDQNGMDVW | V3-21 | J3 | CQVWDSSSDHPWVF | # 2 |
| CLL054 | M | V3-23 | D4-17 | J4 | CAKDINDYGLLAYDYW | V3-21 | J1 | CQVWDSTSDPSYVF |  |
| CLL062 | M | V4-39 | D3-10 | J4 | CANGGGDGEYDYW | V3-21 | J3 | CQVWDSSSDHPWVF |  |
| CLL162 | M | V3-48 | D1-14 | J4 | CARGVPRPHW | V3-21 | J3 | CQVWDSSSDHPWVF |  |
| CLL306 | U | V1-46 | D3-22 | J4 | CARDLYYYDSSGYYSGFFDYW | V3-21 | J3 | CQVWDSSSDQPWVF |  |
| CLL350 | U | V1-69 | D3-16 | J3 | CARDVVDYVWGSYLRAFDIW | V3-21 | J3 | CQVWDSSSDQPWVF |  |
| CLL362 | M | V4-4 | D6-19 | J4 | CARDQVAVAGCFDYW | V3-21 | J1 | CQVWDSSIDPNFVF |  |
| CLL374 | M | V3-48 | D3-10 | J4 | CARDFLTGW | V3-21 | J3 | CQVWDSSSDHPWVF | # 169 |
| CLL381 | M | V3-48 | D6-13 | J4 | CARGAGAGDYW | V3-21 | J3 | CQVWDSSSDQPWVF |  |
| CLL385 | U | V2-26 | D2-21 | J3 | CARDVGGDNSGAFDIW | V3-21 | J1 | CQVWDSSSDLPYVF |  |
| CLL401 | U | V3-21 | D1-14 | J6 | CARDQNTMDVW | V3-21 | J3 | CQVWDSGSDHPWVF | # 2 |

M, mutated (<98% identity to germline; U, unmutated (>98% identity to germline)

**Figures**


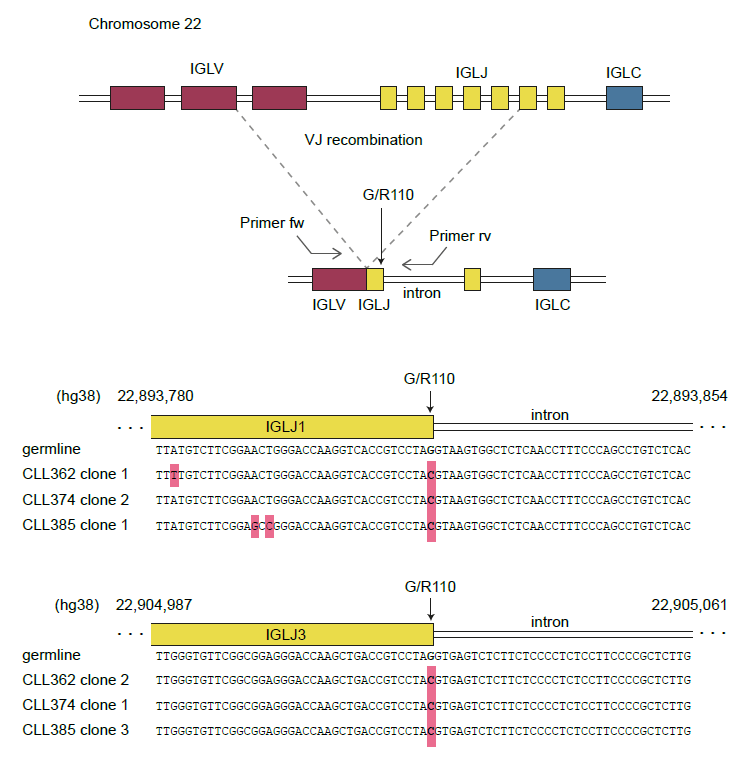


**Supplementary Figure 1: Amplification of the rearranged IGL locus.** Rearranged IGLV-J regions are amplified in a multiplex PCR using IGLV gene specific forward primers and reverse primers annealing at the intron after each IGLJ gene. Alignments of CLL362, CLL374 and CLL385 clones with IGLV3-21R110 and diverging J genes (see also Figure 2) to the germline sequences of IGLJ1 or IGLJ3 and adjacent intron are shown below.

**References**

1. Oberle A, Brandt A, Voigtlaender M, Thiele B, Radloff J, Schulenkorf A, et al. Monitoring multiple myeloma by next-generation sequencing of V(D)J rearrangements from circulating myeloma cells and cell-free myeloma DNA. Haematologica. 2017;102(6):1105-11.

2. Paschold L, Simnica D, Willscher E, Vehreschild MJ, Dutzmann J, Sedding DG, et al. SARS-CoV-2-specific antibody rearrangements in prepandemic immune repertoires of risk cohorts and patients with COVID-19. J Clin Invest. 2021;131(1).

3. Paschold L, Willscher E, Bein J, Vornanen M, Eichenauer DA, Simnica D, et al. Evolutionary clonal trajectories in nodular lymphocyte predominant Hodgkin lymphoma with high transformation risk. Haematologica. 2021.

4. Schieferdecker A, Oberle A, Thiele B, Hofmann F, Göthel M, Miethe S, et al. A transplant "immunome" screening platform defines a targetable epitope fingerprint of multiple myeloma. Blood. 2016;127(25):3202-14.

5. Schliffke S, Akyüz N, Ford CT, Mährle T, Thenhausen T, Krohn-Grimberghe A, et al. Clinical response to ibrutinib is accompanied by normalization of the T-cell environment in CLL-related autoimmune cytopenia. Leukemia. 2016;30(11):2232-4.

6. Schultheiß C, Paschold L, Simnica D, Mohme M, Willscher E, von Wenserski L, et al. Next-Generation Sequencing of T and B Cell Receptor Repertoires from COVID-19 Patients Showed Signatures Associated with Severity of Disease. Immunity. 2020;53(2):442-55.e4.

7. Schultheiß C, Simnica D, Willscher E, Oberle A, Fanchi L, Bonzanni N, et al. Next-Generation Immunosequencing Reveals Pathological T-Cell Architecture in Autoimmune Hepatitis. Hepatology. 2021;73(4):1436-48.

8. Simnica D, Ittrich H, Bockemeyer C, Stein A, Binder M. Targeting the Mutational Landscape of Bystander Cells: Drug-Promoted Blood Cancer From High-Prevalence Pre-neoplasias in Patients on BRAF Inhibitors. Front Oncol. 2020;10:540030.

9. Thiele B, Kloster M, Alawi M, Indenbirken D, Trepel M, Grundhoff A, et al. Next-generation sequencing of peripheral B-lineage cells pinpoints the circulating clonotypic cell pool in multiple myeloma. Blood. 2014;123(23):3618-21.

10. van Dongen JJM, Langerak AW, Brüggemann M, Evans PAS, Hummel M, Lavender FL, et al. Design and standardization of PCR primers and protocols for detection of clonal immunoglobulin and T-cell receptor gene recombinations in suspect lymphoproliferations: Report of the BIOMED-2 Concerted Action BMH4-CT98-3936. Leukemia. 2003;17(12):2257-317.

11. Brüggemann M, Kotrová M, Knecht H, Bartram J, Boudjogrha M, Bystry V, et al. Standardized next-generation sequencing of immunoglobulin and T-cell receptor gene recombinations for MRD marker identification in acute lymphoblastic leukaemia; a EuroClonality-NGS validation study. Leukemia. 2019;33(9):2241-53.

12. Bolotin DA, Poslavsky S, Mitrophanov I, Shugay M, Mamedov IZ, Putintseva EV, et al. MiXCR: software for comprehensive adaptive immunity profiling. Nature Methods. 2015;12(5):380-1.

13. Giudicelli V, Chaume D, Lefranc MP. IMGT/GENE-DB: a comprehensive database for human and mouse immunoglobulin and T cell receptor genes. Nucleic Acids Res. 2005;33(Database issue):D256-61.

14. Bedward M, Eppstein D, Menzel P. Circle Packing: Algorithms to find arrangements of non-overlapping circles. CRAN; 2020. p. <https://github.com/mbedward/packcircles>.

15. Wickham H. ggplot2: Elegant Graphics for Data Analysis. Springer-Verlag New York; 2016. p. <https://ggplot2.tidyverse.org>.
